# Plexin D1 is ubiquitously expressed on tumor vessels and tumor cells in solid malignancies

**DOI:** 10.1186/1471-2407-9-297

**Published:** 2009-08-25

**Authors:** Ilse Roodink, Kiek Verrijp, Jos Raats, William PJ Leenders

**Affiliations:** 1 Dept. of Pathology, Radboud University Nijmegen Medical Centre, P.O. Box 9101, 6500 HB, Nijmegen, The Netherlands; 2 ModiQuest B.V. and Dept. of Biomolecular Chemistry, Nijmegen Centre for Molecular Life Sciences, Heyendaalseweg 135, 6525 AJ, Nijmegen, The Netherlands

## Abstract

**Background:**

Plexin D1 is expressed on both tumor-associated endothelium and malignant cells in a number of clinical brain tumors. Recently we demonstrated that Plexin D1 expression is correlated with tumor invasion level and metastasis in a human melanoma progression series. The objective of this study was to examine whether Plexin D1 might be clinically useful as a pan-tumor vessel and pan-tumor cell target in solid tumors.

**Methods:**

We examined Plexin D1 expression in clinical solid tumors (n = 77) of different origin, a selection of pre-malignant lesions (n = 29) and a variety of non-tumor related tissues (n = 52) by immunohistochemistry. Signals were verified in a selection of tissues via mRNA *in situ *hybridization.

**Results:**

Plexin D1 is abundantly expressed on both activated established tumor vasculature and malignant cells in the majority of primary and metastatic clinical tumors, as well as on macrophages and fibroblasts. Importantly, in non-tumor related tissues Plexin D1 expression is restricted to a subset of, presumably activated, fibroblasts and macrophages.

**Conclusion:**

We demonstrate that Plexin D1 is in general ubiquitously expressed in tumor but not normal vasculature, as well as in malignant cells in a wide range of human tissues. This expression profile highlights Plexin D1 as a potentially valuable therapeutic target in clinical solid tumors, enabling simultaneous targeting of different tumor compartments.

## Background

Interference with a tumor's blood supply is an attractive approach to inhibit tumor growth and dissemination. Thereto, many research focused on targeting the angiogenic process via inhibition of the Vascular endothelial Growth Factor (VEGF-A) pathway. Despite promising results in animal tumor models in which anti-VEGF therapy translates into potent anti-tumor effects [1-3], implementation of these therapies for a number of tumor types in the clinic has now learned that they, either or not in combination with chemotherapy, do increase quality of life or modestly prolong survival [4-7], but lack curative effects [8,9]. This discrepancy may be partly due to the high heterogeneity of the vasculature [10-12]: in established clinical tumors all possible maturation stages may be represented, only a small fraction of which may be susceptible to VEGF inhibition [13]. This situation contrasts that in fast growing animal tumors in which the entire population of tumor vessels may be in a synchronized maturation stage. In addition, we and others described that in organs with intrinsically high vessel densities, tumors and metastases are able to grow in an angiogenesis-independent fashion via co-option of pre-existing blood vessels [14-18]. This provides tumors with a route of escape which makes them (partially) unsusceptible to anti-angiogenic compounds. Even more, anti-angiogenesis may drive a shift in brain tumors from an angiogenic to a co-opting phenotype [19-21]. Therefore, vascular targeting therapy in which the existing tumor vascular bed, angiogenic or pre-existent, is attacked with the aim to induce acute tumor-specific coagulation may be an attractive additional approach to deprive a tumor from blood supply. To apply vascular targeting therapies, targetable markers that discriminate tumor vessels from normal vasculature are needed. We previously described that Plexin D1 (PLXND1) could be such a target [22].

PLXND1 belongs to a family of large transmembrane proteins that are receptors for neuropilins and semaphorins [23,24]. Plexins are involved in regulation of axonal patterning during embryonic development [25-28]. Apart from neuronal cells, PLXND1 is also expressed by vascular endothelial cells during embryogenesis [29] and is of pivotal importance for vascular patterning, as illustrated by the fact that PLXND1 knock-down in mice and zebrafish results in abnormal development of the cardiovascular system [30-32].

We previously demonstrated that PLXND1 is also specifically expressed on vascular endothelium during tumor-associated angiogenesis in a mouse xenograft model of cerebral melanoma metastasis and in a number of human brain tumors, both of primary and metastatic origin [22]. Importantly, expression of this protein was also found on tumor cells in these tumors [22], and this expression correlates with malignancy grade in a human melanoma progression series: whereas PLXND1 is abundantly expressed in both invasive primary and disseminated melanomas, both in the vasculature and in tumor cells, its expression was absent in benign melanocytic lesions and melanomas *in situ*, except for expression on macrophages and fibroblasts [33].

PLXND1 contains in its intracellular domain consensus Rac/RhoA signalling motifs [29], suggestive of a role in cytoskeletal rearrangements and cell motility, processes which are fundamental for both tumor angiogenesis and metastasis. PLXND1 may thus be functionally involved in tumor development in multiple ways.

The expression profile of PLXND1 suggests that it may be a valuable tumor target for established solid tumors, allowing simultaneous targeting of different tumor compartments, i.e. vessels and tumor cells. To examine whether PLXND1 might be clinically useful as a pan-tumor vessel and pan-tumor cell target in solid tumors we analyzed PLXND1 expression in a wide range of human tumors of different origin, various pre-malignant and non-tumor related tissues by immunohistochemistry and mRNA *in situ *hybridization.

## Methods

### Tissue samples

Primary and metastatic tumor tissues of different origin (n = 77), among them 15 paired samples of human primary and metastatic lesions and various pre-malignant lesions (n = 29), were selected from the archives of the Department of Pathology and Radiology of the Radboud University Nijmegen Medical Centre. Furthermore, non-tumor related tissues (n = 52) were obtained. The study was performed according to the guidelines of the Code for proper secondary use of human tissue in the Netherlands (Version 2002, Federation of Biomedical Scientific Societies, http://www.federa.org).

### Immunohistochemistry

After deparaffinization and blocking of endogenous peroxidase activity, antigen retrieval was performed by treatment with pronase according to standard protocols [33]. Non-specific binding sites were blocked by incubation with 20% normal horse serum. Slides were incubated for 1 hr with single domain antibody A12, which was previously selected against a PLXND1-specific peptide [22]. A12 was detected by sequential incubations with the mouse anti-VSV-G P5D4, biotinylated anti-mouse IgG (Vector, Burlingame, CA), and avidin-biotin peroxidase complex (Vector). Peroxidase was visualized by the 3-amino-9-ethylcarbazole (ScyTek, Logan, UT) peroxidase reaction, with haematoxylin as counterstain. All incubations were performed at room temperature. Blood vessel origin was confirmed by endothelial stainings on serial sections with anti-human CD31 antibody (DAKO, Glostrup, Denmark).

In a selection of tissues, macrophage identity was confirmed by double staining for PLXND1 and CD68. In short, the above mentioned avidin-biotin peroxidase procedure was used to detect PLXND1 via rabbit anti-VSV-G antiserum (Sigma Chemical Co., Zwijndrecht, The Netherlands). Following visualization, avidin-biotin was blocked according to standard protocols. Slides were successively incubated with normal horse serum, mouse anti-human CD68 antibody (DAKO) overnight at 4°C, biotinylated anti-mouse IgG (Vector) and avidin-biotin alkaline phosphatase (AP) complex (Vector) at RT. AP was visualized with a mixture of naphthol phosphate (Sigma), levamisole (Sigma), and Fast Blue (Sigma).

### PLXND1 mRNA In Situ Hybridization

To explore whether the PLXND1 transcript is also specifically present in malignant tissues, we performed mRNA *in situ *hybridizations on 28 of the 158 paraffin-embedded tissues analyzed by immunohistochemistry (malignant lesions, n = 15; non-tumor related samples, n = 13) as previously described [22]. In brief, digoxigenin-labelled sense and antisense human PLXND1 RNA probes, located in the 3'-untranslated region, were generated by *in vitro *transcription from a PLXND1 PCR product which was flanked by T7 and T3 promoters as described [29]. Following deparaffinization, 4 μm tumor sections were treated with proteinase K (Roche, Almere, The Netherlands) at 37°C for 15 minutes and post-fixed in formaldehyde. Non-specific binding sites were blocked by incubation with acetic anhydride (Sigma) at room temperature. Tissues were hybridized with digoxigenin-labelled RNA probes at 63°C with 200 ng/ml probe. Single stranded non-hybridized RNA was degraded with RNAse T1 (2 units/ml) at 37°C for 30 minutes. After subsequent pre-incubation with normal sheep serum, the slides were incubated with alkaline phosphatase-conjugated sheep anti-digoxigenin antibody (Roche) at room temperature for 1 hour. Alkaline phosphatase was visualized using nitro blue tetrazolium (NBT; Roche) and 5-bromo-4-chloro-3-indolyl phosphate (BCIP; Roche) as substrate with nuclear fast red as counterstain. Specificity of hybridizations was verified by performing control hybridizations with sense probe.

## Results

**Figure 1:**
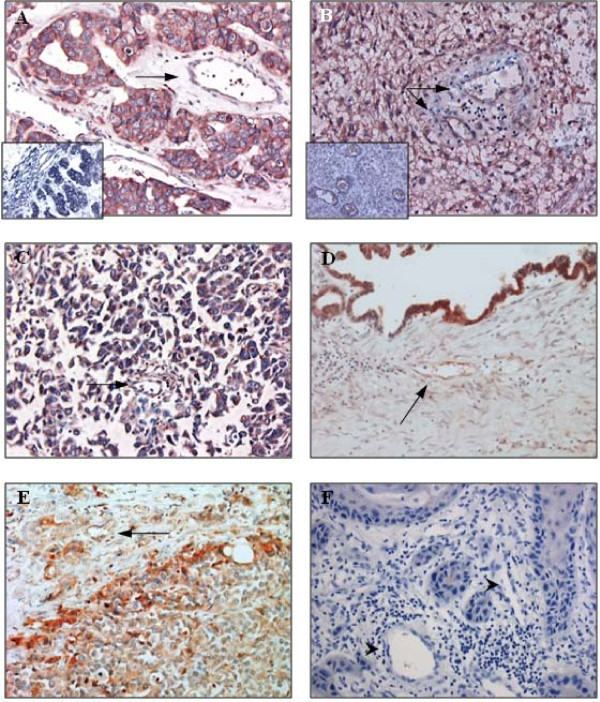
**PLXND1 expression in representative clinical tumor samples**. Immunohistochemical analyses using a single domain antibody against PLXND1 (Magnification ×200). PLXND1 is abundantly expressed in adenocarcinoma brain metastases (A), glioblastomas multiforme (B), neuro-endocrine lung tumors (C), an ovarian adenocarcinoma (D), and prostatic urothelial cell carcinomas (E). The arrows point at PLXND1-positive vasculature. PLXND1 is absent in both tumor vasculature (arrowheads) and tumor cells in vulvar squamous cell carcinomas (F). The insets in A and B show corresponding PLXND1 mRNA *in situ *hybridization analyses (Magnification ×200).

**Figure 2:**
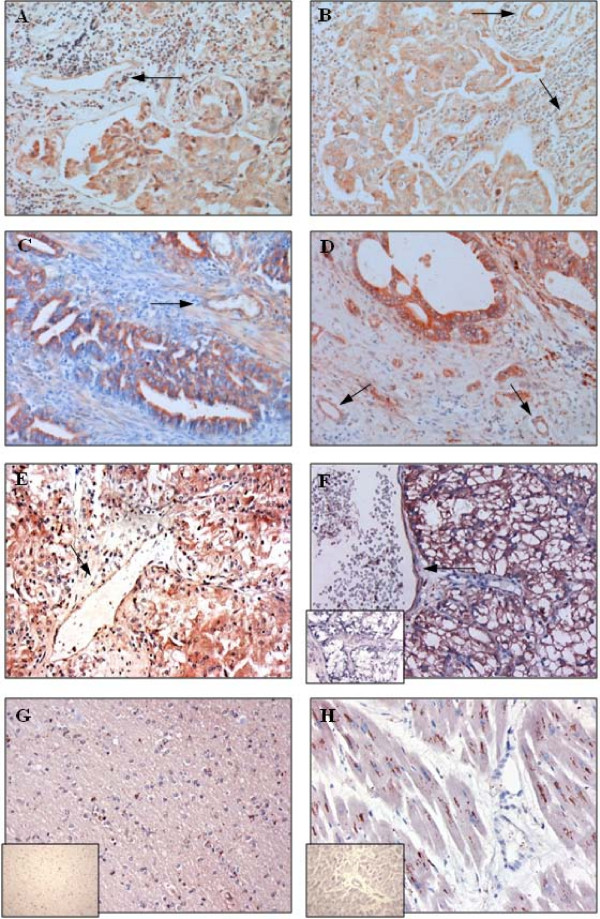
**PLXND1 expression in representative clinical (tumor) samples**. Immunohistochemical analyses using a single domain antibody against PLXND1 (Magnification ×200). PLXND1 is expressed at high levels in primary ductal breast carcinomas (A) and corresponding lymph node metastases (B), colon adenocarcinomas (C) and corresponding liver metastases (D) and a renal cell carcinoma (E) and corresponding brain metastasis (F). The arrows point at PLXND1-expressing tumor vessels. PLXND1 is not detected in normal human cerebral cortex (G) and heart (H) tissue samples. The insets in F, G and H show corresponding PLXND1 mRNA *in situ *hybridization analyses (Magnification ×200).

To explore whether PLXND1 may be clinically useful as a pan-tumor endothelium and a pan-tumor cell target we examined the expression profile of this protein in a large variety of human tissue samples. As summarized in Table 1, PLXND1 is abundantly expressed in both the vasculature and malignant cells in the majority of clinical tumors, whereas in pre-malignant lesions the protein is present at lower levels or, like in non-tumor related tissues, almost completely absent (Table 2). Figure 1 shows vascular (arrows) and tumor cell expression of PLXND1 in representative samples of brain metastasis of adenocarcinoma (A), glioblastoma multiforme (B), neuro-endocrine lung tumor (C), ovarian adenocarcinoma (D), and prostatic urothelial cell carcinoma (E). The insets in A and B show corresponding *in situ *hybridization signals. Vascular and tumor cell-associated PLXND1 expression was absent in 3 out of 5 medullary breast carcinomas, one out of 5 cervical squamous cell carcinomas, and all examined vulvar squamous cell carcinomas (Table 1). A representative sample of vulvar squamous cell carcinoma is shown in figure 1F. In addition, two low grade astrocytomas showed infrequent vascular and tumor cell-associated PLXND1 expression (Table 1). As shown in figure 2A through F no significant differences in staining pattern and intensity of vascular structures (arrows) and malignant cells could be observed between primary ductal breast carcinoma (A) and a corresponding lymph node metastasis (B), colon adenocarcinoma (C) and a corresponding liver metastasis (D) and renal cell carcinoma (E) and a corresponding brain metastasis (F, the inset shows corresponding *in situ *hybridization signal).

**Table 1:** Plexin D1 expression in solid malignancies.

Malignant tissue	PLXND1 expression				Remarks
		
	*Endothelial cells*	*Tumor cells*	*Subset of macrophages*	*Subset of fibroblasts*	
					

***Paired samples***					

Medullary breast carcinoma (n = 1)	-	-	+	+	Plasma cells also positive

Lymph node metastasis (n = 1)	Subset	-	+	n.d.	

					

Ductal breast carcinoma (n = 8)	+	+	+	+	Some plasma cells also positive

Lymph node metastasis (n = 8)	+	+	+	+	

					

Adenocarcinoma of colon (n = 4)	+	+	+	+	

Liver metastasis (n = 4)	+	+	+	+	

					

Alveolar soft part sarcoma of femur (n = 1)	+	+	n.d.	n.d.	

Brain metastasis (n = 1)	+	+	n.d.	n.d.	

					

Renal cell carcinoma (n = 1)	+	+	n.d.	n.d.	

Brain metastasis (n = 1)	+	+	n.d.	n.d.	

					

***Non-paired samples***					

Adenocarcinoma of coecum (n = 1)	+	+	+	-	

Adenocarcinoma of oesophagus (n = 1)	+	+	+	-	

Adenocarcinoma of ovary (n = 1)	Subset	+	+	+	

Adenocarcinoma of prostate (n = 1)	+	+	-	-	

Adenocarcinoma of rectum (n = 5)	+	+	+	+	

Brain metastasis of adenocarcinoma (n = 4)	+	+	n.d.	n.d.	

Liver metastasis of adenocarcinoma colon (n = 8)	+	+	+	+	

Ovary metastasis of adenocarcinoma colon (n = 1)	-	+	-	-	

					

Low grade astrocytoma (n = 2)	Subset	Subset	n.d.	n.d.	

Glioblastoma Multiforme (n = 3)	+	+	n.d.	n.d.	

Medulloblastoma (n = 1)	+	+	n.d.	n.d.	

					

Neuro-endocrine tumor of lung (n = 2)	+	+	+	n.d.	

					

Medullary breast carcinoma of (n = 4)	+	+	+	+	2 samples negative vessels and tumor cells

Lymph node metastasis of ductal breast carcinoma (n = 1)	Subset	+	-	-	

					

Squamous cell carcinoma of cervix (n = 5)	Subset	+	+	-	1 sample negative vessels and tumor cells

Squamous cell carcinoma of vulva (n = 5)	-	-	+	+	

					

Urothelial cell carcinoma of prostate (n = 2)	+	+	+	-	

+, corresponding cell type expresses PLXND1

-, PLXND1 expression is absent in corresponding cell type

n.d., corresponding cell type is not detected

**Table 2:** Plexin D1 expression in pre-malignant and non-tumor related tissues.

Tissue	PLXND1 expression			Remarks
		
	*Endothelial cells*	*Subsets of macrophages*	*Subsets of fibroblasts*	
				

***Pre-malignant samples***				

Ductal carcinoma *in situ *of breast (n = 5)	+	+	+	Weak staining of tumor cells

Lobular carcinoma *in situ *of breast (n = 3)	Weak	+	+	Weak staining of tumor cells

Vulvar intraepithelial neoplasia (VIN)				

Classic VIN III, HPV negative (n = 5)	-	+	+	

Classic VIN III, HPV positive (n = 8)	-	+	+	

Differentiated VIN III (n = 8)	-	+	n.d.	

				

***Non-tumor related samples***				

Bladder (n = 1)	-	+	-	

Blood vessel				

Atherosclerosis (n = 6)	-	+	-	

Fatty streaks (n = 1)	-	+	-	

Bone marrow (n = 2)	n.d.	-	-	

Brain				

Cortex (n = 1)	-	n.d.	n.d.	Some neurons perinuclear staining

Alzheimer and CAA (n = 1)	-	n.d	n.d.	

Multiple Sclerosis (n = 2)	-	n.d	n.d.	

Breast				

Normal breast (n = 2)	-	-	-	Some epithelial cells perinuclear staining

Ductal hyperplasia (n = 1)	-	+	-	Focal epithelial cells perinuclear staining

Endometrium				

Proliferation phase (n = 5)	Subset (n = 3)	+	n.d.	

Secretion phase (n = 4)	-	+	n.d.	

Secretion/menstruation phase (n = 1)	-	+	n.d.	

Endometriosis interna (n = 1)	-	+	n.d.	

Heart (n = 1)	-	n.d	n.d	Myocytes perinuclear staining

Large intestine (n = 1)	-	+	+	Some epithelial cells perinuclear staining

Liver (n = 1)	-	+	n.d.	Some hepatocytes perinuclear staining

Lung (n = 2)	-	+	n.d.	

Oesophagus (n = 1)	-	+	-	

Pancreas (n = 1)	-	n.d	n.d.	Perinuclear staining Islets of Langerhans

Pituitary gland (n = 1)	-	n.d.	n.d.	Some epithelial cells perinuclear staining

Prostate (n = 1)	-	-	n.d.	Some epithelial cells perinuclear staining

Small intestine (n = 1)	-	+	+	Some epithelial cells perinuclear staining

Spleen (n = 1)	-	+	n.d.	

Thyroid gland (n = 1)	-	+	n.d.	Some epithelial cells perinuclear staining

Vulva				

Normal vulva (n = 6)	-	+	+	

Lichen sclerosus (n = 6)	-	+	+	

+, corresponding cell type expresses PLXND1

-, PLXND1 expression is absent in corresponding cell type

n.d., corresponding cell type is not detected

To examine whether PLXND1 is expressed on angiogenic vessels under physiological conditions, we also investigated expression in endometrium. In 3 out of 5 proliferative phase endometria some vessels stained positive for PLXND1 (Table 2). Figure 2G and 2H show lack of PLXND1 protein and transcript (insets) in normal human cerebral cortex and heart tissue. The granular staining pattern in cerebral neurons (G) and cardiac myocytes (H) is presumably due to aspecific binding of single domain antibodies to lipofuscine as this is observed with other non-related single domain antibodies too (not shown). Apart from tumor-associated endothelium and tumor cells, PLXND1 expression was also observed in subsets of fibroblast- and macrophage-like cells in both tumor samples and pre-malignant and non-tumor related tissues. Identity of macrophages was confirmed by double staining a selection of analyzed tissues for PLXND1 and CD68 (not shown).

## Discussion

In previous work we showed that PLXND1 is expressed in endothelial cells during developmental and tumor-associated angiogenesis [22,29]. Besides vascular expression, high PLXND1 expression was also found on tumor cells in cerebral melanoma metastases. Whereas a role of PLXND1 in vessel patterning during development is well established [30-32], the functional consequences of PLXND1 expression on tumor cells and vessels are less clear. We recently demonstrated that PLXND1 expression is correlated with tumor invasion and metastasis in a human melanoma progression series [33]. However, the PLXND1 ligands Semaphorin 3E and 4A inhibit, rather than promote, (tumor) angiogenesis [33,34]. Moreover, Semaphorin 3E even exhibits anti-tumor and anti-metastatic properties [33,35].

Here we show that PLXND1 is expressed at high levels on activated established tumor vasculature in a variety of primary and metastatic human malignancies, whereas in non-tumor related tissues PLXND1 expression is restricted to a subset of, presumably activated, fibroblasts and macrophages. These results are in agreement with our previous observations in clinical brain tumors of different origin [22] and a series of cutaneous melanocytic lesions representing different stages of melanoma progression [33]. So, for subsets of tumor types in which vessel activation has occurred, PLXND1 may be a valuable candidate protein for vascular targeting approaches. Indeed, the anti-PLXND1 single domain antibody A12 homes to and accumulates in tumor vessels [22].

Successful vascular targeting has also been achieved with agents directed against molecules of which expression is restricted to vessels in early stages of angiogenesis. Examples are the L19 single chain antibody, directed against the ED-B fragment of fibronectin which targets vasculature in actively growing tumors [36], whereas this single chain antibody is unable to detect quiescent endothelium in low grade malignancies [37]. Targeted radiotherapy with radiolabeled RGD peptides, recognizing integrin αvβ3 on newly formed endothelial cells, led to reduced growth of xenografts in mouse models of cancer [38-40]. Furthermore, chimeric proteins, consisting of antibodies against the tumor vessel marker vascular cell adhesion molecule 1 (VCAM-1), fused to soluble Tissue Factor, induced tumor specific blood clotting, tumor necrosis and growth delay in different xenograft models [41].

Due to vessel heterogeneity in tumors [10-12,42] it is unlikely that one single marker will behave as a targetable pan-tumor-endothelial antigen, but appropriate mixtures of different tumor vessel targeting agents, including anti-PLXND1 antibodies, may allow specific targeting of the majority of tumor vessels. For instance, to effectively starve tumors like low grade gliomas, which (partly) thrive on quiescent vasculature, targeting agents that recognize co-opted vessels in infiltrative tumor areas need to be developed. It remains to be seen whether such targets can be identified and, if so, whether such strategy holds promise for treatment of brain tumors, as it will include some toxicity for interspersed normal brain in infiltrative tumor areas.

Apart from vascular PLXND1 expression in tumors, the protein is also abundantly expressed on tumor cells in a wide range of clinical solid tumors which reinforces this membrane protein as a tumor target, since it allows simultaneous targeting of different tumor compartments with one compound. Since all tumor metastases tested, except for one lymph node metastasis of a medullary breast carcinoma, expressed high levels of this protein, both on vessels and tumor cells, a PLXND1-directed seek-and-destroy strategy may therefore indeed be feasible.

Despite its abundant expression in many different tumor types PLXND1 was not expressed on tumor cells and vessels in a subset of medullary breast carcinomas. Interestingly, these relatively rare tumors generally have a favorable prognosis [43]. It is tempting to speculate that PLXND1 expression is generally correlated with increased malignancy grade. The only other tumor type in our series which lacked vascular and tumor cell-associated PLXND1 expression was vulvar squamous cell carcinoma. Because the increased microvessel density in these tumors suggests angiogenesis [44], the lack of PLXND1 on vessels in these tumors was unexpected [22]. Whether a lack of PLXND1 expression on these vessels is related to a more mature state can only be speculated upon. Physiological angiogenesis in proliferative phase endometria has been described to occur mainly in a non-sprouting fashion via vessel elongation [45] which may explain the appearance of vascular PLXND1 in only a small subset of vessels in such tissue.

## Conclusion

We demonstrated that PLXND1 is in general ubiquitously expressed in tumor but not normal vasculature, as well as in malignant cells in a wide range of human (tumor) tissues. This expression pattern warrants further investigation towards PLXND1 as a therapeutic target in oncology.

## Competing interests

The authors declare that they have no competing interests.

## Authors' contributions

IR performed the immunohistochemical analyses. KV carried out mRNA *in situ *hybridization and assisted in the interpretation of the results. JR participated in the design of the study and provided general support. IR and WPJL were responsible for experimental design, interpretation of the results and writing the manuscript. All authors read and approved the final manuscript.

## Pre-publication history

The pre-publication history for this paper can be accessed here:

http://www.biomedcentral.com/1471-2407/9/297/prepub
